# The Chorion Proteome of *Diaphorina citri*, the Vector of Huanglongbing Disease in Citrus

**DOI:** 10.3390/insects12110959

**Published:** 2021-10-21

**Authors:** Yulica Santos-Ortega, Nabil Killiny

**Affiliations:** 1 Department of Plant Pathology, Citrus Research and Education Center, IFAS, University of Florida, 700 Experiment Station Road, Lake Alfred, FL 33850, USA; yulica.santosortega@usm.edu; 2 Department of Biological Environmental and Earth Sciences, Discipline: Cell and Molecular Biology, The University of Southern Mississippi, 118 College Drive, Hattiesburg, MS 39406, USA

**Keywords:** chorion, hatching, proteomics, *Diaphorina* *citri*, LC-MS/MS

## Abstract

**Simple Summary:**

Huanglongbing is currently the most devastating disease of citrus worldwide. It is believed that controlling the vector, *Diaphorina citri*, is the most important and effective method of disease management. To achieve this goal, we have employed RNA interference (RNAi), a promising technique for controlling *D. citri*. Our present strategy is to target *D. citri* egg hatching. By reducing the rate of egg hatching, we will reduce the vector population and subsequently reduce the spread of disease. In order to interfere with the egg hatching mechanism, we studied the chorion proteome to identify potential gene candidates for RNAi.

**Abstract:**

Nowadays, the Asian citrus psyllid, *Diaphorina citri* (Kuwayama) (Hemiptera: Liviidae) is considered the most devastating pest of citrus because it transmits “*Candidatus* Liberibacter asiaticus”, the putative causal agent of huanglongbing (HLB) or citrus greening. Controlling the vector is the main strategy used to mitigate HLB. Targeting *D. citri* at the very early stages of its development may offer an effective control strategy. Identifying chorion proteins will contribute to a better understanding of embryo development and egg hatching and thus could lead to valuable targets to better control psyllid populations. Herein, we analyze the chorion proteins of *D. citri.* Mass spectrometry-based bottom-up/shotgun proteomics and databases were queried to achieve protein identification. Fifty-one proteins were identified in *D. citri* chorion. The *D. citri* chorion proteins were divided into eight categories according to their biological or molecular function: *i—*enzymes (25%); *ii—*binding proteins (10%); *iii—*structural proteins (8%); *iv—*homeostasis-related proteins, mostly vitellogenins (8%); *v—*proteins related to gene expression (6%); *vi—*immune system proteins (6%); *vii—*other proteins (16%); and *viii—*uncharacterized proteins (21%). The composition of the chorion proteome suggested that the hatching rate could be reduced by silencing chorion-related genes. The proteomic analysis of *D. citri* chorion tissue allowed us to identify its proteins, providing promising new targets for *D. citri* control through RNA interference technology.

## 1. Introduction

The Asian citrus psyllid, *Diaphorina citri* (Kuwayama) (Hemiptera: Liviidae), is a sap-sucking hemipteran that serves as the main vector for “*Candidatus* Liberibacter asiaticus”, the putative causal agent of citrus huanglongbing (HLB) [1,2]. Once inoculated into the host tree, the bacterium multiplies and initiates a cascade of host plant responses that leads to callose deposition in leaves [3,4], starch accumulation, asymmetrical leaf mottling [5], and root loss [6]. HLB is rapidly transmitted by *D. citri* as it feeds on the phloem sap of Rutaceae species, which include *Citrus* and its relatives [1]. *D. citri* is the insect vector of “*Ca*. L. asiaticus” in Asia, Brazil, and the USA [7,8]. Severe economic losses result from the infestations with *D. citri* because it colonizes the new shoots, transmitting “*Ca*. L. asiaticus” to trees and causing damage to foliage. Trees infected with “*Ca*. L. asiaticus” are short-lived, have reduced yields, and produce smaller, lopsided fruit with poor quality juice [9,10,11,12].

Mitigation of HLB has been achieved primarily through the chemical control of *D. citri*, but it has failed to contain the spread of the disease in the US. Chemical pesticide use for *D. citri* control has led to reports of insecticide resistance [13,14] and is non-specific, and off-target effects may harm beneficial insects such as honeybees. Furthermore, most insecticides have negative environmental impacts, and applications are difficult to coordinate among growers [15]. Numerous non-chemical control strategies have been attempted with some degree of success, including kaolin clay treatments [16], heat treatments [17], enhanced nutritional programs [17,18,19], and biological control [20] (see reviews [21,22]), but nearly 100% of Florida citrus groves are currently affected by HLB disease.

A relatively modern biotechnological approach known as RNA interference (RNAi) has shown promising results for “silencing” genes in *D. citri* to study functional genomics. The sequencing of the *D. citri* genome and the resulting transcriptome has allowed the discovery of many diverse predicted proteins [23,24,25]. However, the new challenge is to identify the functions and roles these genes play in the lifecycle of *D. citri* and find those that can be exploited against it. RNAi techniques have been utilized extensively to study potential control targets. Double-stranded RNA (dsRNA) can be applied to adult psyllids topically [26], and through an artificial diet [27,28,29], while nymphs can be treated easily through topical feeding [30] or soaking in dsRNA solutions [31]. RNAi technologies were recently reviewed [32]. We targeted genes implicated in the development and metamorphosis of *D. citri* (abnormal wing disc, muscle protein 20) [31,33,34], gender ratios (boule and transformer-2 homologues) [27,28], metabolism (sucrose hydrolase) [35], and insecticide resistance (cytochrome P450, acetylcholinesterases, glutathione S-transferase) [26,29,30,36]. So far, RNAi treatments to *D. citri* have been restricted to the laboratory.

The order Hemiptera, which includes phloem-feeding aphids, psyllids, whiteflies and leafhoppers, pass through several life stages, from egg to nymphs to adult, without a pupal stage. *D. citri* females lay 400–600 eggs in the soft new flush of citrus or other host plants, which incubate at ambient temperatures for about 4 d [37]. Nymphs pass through five instar stages before molting into the adult form 12–13 d later [37]. During incubation, the chorion (eggshell) protects insect embryos from the environment [38]. During the later stages of oogenesis, the chorion matrix is secreted between the oocyte and overlaying somatic follicle cells [39]. Its multilayered structure performs a variety of specialized functions such as providing substrate attachment, resistance to acid or alkalis, preventing water loss, and allowing gas exchange throughout development [39,40]. The differences in ecological environment and, specifically, oviposition substrates have determined through evolution the chorion morphology, organization, and composition among different species [41]. Chorion proteins are the components that play a pivotal role in chorion assembling and stabilization and ensure the success of embryo development after oviposition [42].

To our knowledge, the very early developmental stages, including egg hatching and embryo development, have not been fully exploited to control *D. citri*, with only one report of embryo suppression using CRISPR gene knock-out [43,44]. To target egg production by RNAi, information about the proteins that function in chorion formation would be needed. Therefore, we used proteomic approaches to aid in the identification of *D. citri* chorion proteins to provide new targets to control psyllids from their earliest stages. To achieve this goal, we employed mass spectrometry-based bottom-up/shotgun proteomics in conjunction with the Uniprot database to identify the *D. citri* chorion proteins.

## 2. Materials and Methods

### 2.1. Insect Colonies

The laboratory colony of *D. citri* was reared continuously on healthy *Citrus macrophylla* seedlings 3–6 months old. The seedlings and *D. citri* were maintained in a controlled-temperature growth room with a 16:8 h L:D photoperiod, 27 ± 2 °C, and 60 ± 5% relative humidity. This facility is located at the Citrus Research and Education Center in Lake Alfred (28.092783° N, 81.72307° E), Florida.

### 2.2. Chorion Collection and Sample Preparation

**Figure 1:**
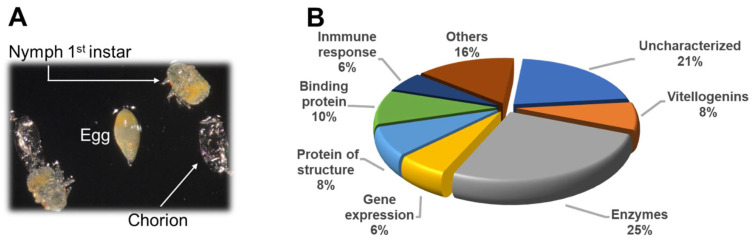
Chorion proteins of *Diaphorina citri*. (**A**) Egg hatching and recovery of chorion from the artificial medium. (**B**) Putative biological functions of *D. citri* eggshell proteins. The 51 *D. citri* chorion proteins were classified according to their biological activities using the UniProt browser for gene ontology terms and annotations and the SMART domain http://smart.embl-heidelberg.de/ accessed on 19 September 2021.

Egg collection was carried out in an artificial medium created specifically for egg hatching from *D. citri* in our laboratory [45] (Figure 1A). Briefly, the medium was comprised of 25% of sucrose, 1% yeast, sterilized water (10 mL), and 0.56 g of unflavored gelatin. The leaves of *Citrus macrophylla* plants containing psyllid eggs were collected and washed for 5 min in 0.03% bleach (1% of a commercial product), followed by three rinses in sterile water. Afterwards, the eggs were carefully removed from the leaves using a modified dissecting needle (bent slightly at the tip), immediately placed on the artificial medium, and kept at ambient temperature (25 °C). Following the hatching of the first instar *D. citri* nymphs, approximately 420 choria were recovered. The choria were homogenized with a pestle in a solution of 0.1 M of ammonium acetate (NH_4_Ac) and were briefly centrifuged to discard debris. The sample was transferred to a new microcentrifuge tube, and the determination of protein concentration was performed using the Bicinchoninic acid method (Pierce^TM^ BCA Protein Assay Kit, ThermoFisher Scientific, Waltham, MA, USA). The sample was stored at −80 °C until analysis.

### 2.3. Reduction/Alkylation and In-Solution Digestion of Chorion Proteins

The chorion sample (25 µL initial volume) in 0.1 M of NH_4_Ac was reduced with 5 µL of 200 mM dithiothreitol (DTT) at 95 °C for 5 min and at 55 °C for 45 min. The sample was alkylated with 4 µL of 1 M chloroacetamide (CAA) at 25 °C for 45 min in the darkness, and the reaction was stopped with 20 µL of 200 mM DTT at 25 °C for 45 min. Trypsin solution was prepared in ammonium bicarbonate (NH_4_HCO_3_) for tryptic digestion of the sample in a 1:50 trypsin:protein ratio at 37 °C overnight (In-Solution Tryptic Digestion Kit, Thermo Scientific, Waltham, MA, USA) [46].

### 2.4. Desalting of Digested Protein

The digested chorion sample was desalted with micro ZipTip C18 (Millipore, St. Louis, MO, USA) by equilibrating the tip in 100% acetonitrile (ACN) (1 × 10 µL), 50% ACN/50% of 0.1% trifluoroacetic acid (TFA) (1 × 10 µL), and 0.1% TFA (3 × 10 µL). The sample was loaded into the ZipTip by pipetting the sample 10 times and washing with 0.1% TFA (10 × 10 µL) before eluting. The protein fragments were eluted with 80% ACN/0.1% TFA and dried in a SpeedVac.

### 2.5. LC-MS/MS Mass Spectrometry Analysis

LC-MS/MS was carried out at the Interdisciplinary Center for Biotechnological Research, University of Florida, Gainesville, FL. The digested sample was resuspended in 0.1% formic acid, and mass spectrometry was performed on an EASY-nLC^TM^ 1200 ultra-high-performance liquid chromatography system (Thermo Fisher Scientific, Waltham, MA, USA) connected to an Orbitrap Fusion^TM^ Tribrid^TM^ instrument equipped with a nano-electrospray source (Thermo Fisher Scientific, Waltham, MA, USA). The digested sample was loaded into a C18 trapping column (Acclaim^TM^ PepMap^TM^ 100, 75 µm inner diameter × 2 cm length, 3 µm particle size, and 100 Å pore size) and eluted with a C18 analytical column (Acclaim^TM^ PepMap^TM^ 100, 75 µm inter diameter × 15 cm length, 2 µm particle size, and 100 Å pore size) at a flow rate of 250 nL/min. The separation was using solvent A (0.1% formic acid in water) and solvent B (0.1% formic acid and 80% acetonitrile) as the mobile phases, increasing the gradient as follows: 2–35% of solvent B over 0–140 min; 35–80% of solvent B over 40–45 min, 80–98% of solvent B over 45–46 min, and kept at 98% of solvent B until 60 min [47]. The full MS1 scan (*m/z* 350–2000) was performed on the Orbitrap Fusion with a resolution of 120,000 at *m/z* 200 [48]. The automatic gain control (AGC) target was 2 × 10^5^, with 50 ms as the maximum injection time. Monoisotopic precursor selection (MIPS) was enforced to filter for peptides. Peptides bearing ^+^2–6 charges were selected with an intensity threshold of 1 × 10^4^. Dynamic exclusion of 15 s was used to prevent resampling the high abundance peptides. The top speed method was used for data-dependent acquisition within a cycle of 3 s. The MS/MS was carried out in the ion trap, with a quadrupole isolation window of 1.3 Da. Fragmentation of the selected peptides by collision-induced dissociation (CID) was done at 35% of normalized collision energy. MS^2^ spectra were detected in the linear ion trap with the AGC target as 1e4 and the maximum injection time as 35 ms.

### 2.6. Database Searches

MS/MS data were analyzed using Mascot version 2.7.0.1 (Matrix Science, London, UK) by searching against the UnitProt-Diaphorina_citri_20200316 database (unknown version, 22,073 entries) (https://www.uniprot.org/proteomes/UP000079169/ accessed on 20 September 2021), assuming digestion with trypsin. Fragment ion mass tolerance was set at 1.00 Da and parent ion tolerance at 10 ppm 0^+18^ of pyrrolysine and carbamidomethyl of cysteine, specified in Mascot as fixed modifications [46,47]. Gln->pyro-Gln of the N-terminus, deamidate of asparagine and glutamine, and oxidation of methionine were specified in Mascot as variable modifications. Peptide identifications were accepted if they could be established at greater than 95.0% probability by the Scaffold Local FDR algorithm. Protein identifications were accepted if they could be established at greater than 99.0% probability and contained at least two identified peptides. Protein probabilities were assigned by the Protein Prophet algorithm [49]. Further, MS/MS-based peptide and protein identification was validated using Scaffold version 4.10.0 (Proteome Software Inc., Portland, OR, USA).

## 3. Results and Discussion

The chorion covers the whole surface area of the insect eggs to impair water loss, allow gas exchange during development, and protect the eggs from the maternal body [50]. The chorion is a highly specialized, multilayered barrier that allows for the fertilization of the oocyte and adjusts its permeability to allow optimal conditions for embryo development. These conditions vary dramatically depending on the insect species and its ecology, i.e., whether eggs are laid on plant leaves, in soil, above or below ground, or in water. Chorion composition, structure, and physical properties determine its permeability, restricting the egg’s development to specific environments [51]. Chorion is composed of proteinaceous and organic molecules [52] that can be identified, in part, through proteomics analysis [51,53,54].

In the current study, we identified 51 proteins (Table 1) in *D. citri* chorion. The proteins were categorized by their biological or molecular function, as indicated in Figure 1B. The *D. citri* chorion proteins putatively identified were divided into eight categories: (i) enzymes such as hydrolases, transferase, and enzymes related to GTPase and glycosidase activity; (ii) binding proteins; (iii) structural proteins; (iv) homeostasis-related proteins (mostly vitellogenins); (v) proteins related to gene expression; (vi) immune system proteins; (vii) other proteins; and (viii) uncharacterized proteins.

### 3.1. Enzymes

The largest group found in the chorion proteins were enzymes (25%), and these consisted largely of hydrolases such as the probable chitinase 10 GH18 family (306 kDa), the endochitinase GH18 family (68 kDa), chitooligosaccharidolytic β-N-acetylglucosaminidase-like (38 kDa), and alpha galactosidase (17 kDa). Several glycoside hydrolases were identified in *D. citri* chorion (chitinase or endo-N-acetyl-β-D-glucosaminidase (ENGase), chitinase-like lectins (chi-lectins/proteins (CLPs), alpha mannosidase, and alpha-glucosidase). Although the function of these enzymes in insect chorion is not completely understood, in general, they are involved in chitin biosynthesis and the degradation of insect anatomical structures, such as the trachea, mouthparts, and cuticle [55]. On the other hand, the presence of carbohydrates in chorion has been involved in fertilization as a putative ligand for sperm enzymes [56].

Three proteins were characterized as peptidases, including carboxypeptidase D-like (145 kDa), aminopeptidase (92 kDa), and an uncharacterized 91 kDa protein. We previously detected both a carboxypeptidase and a hypothetical aminopeptidase in the cytosolic protein fraction of adult *D. citri* [46] that are likely involved in general proteolysis. Similarly, in the saliva proteins of *D. citri*, Yu and Killiny [47] detected a 472.7 kDa protein with histone acetyltransferase activity similar to histone acetyltransferase p300, but its small size in the chorion fraction (30 kDa) suggests a different homology. The remaining enzymes identified included epoxide hydrolase 4-like (46 kDa) and apoptosis-inducing factor-1-mitochondrial (81 kDa), which was assigned as an oxyreductase.

### 3.2. Binding Proteins

Three types of binding proteins (10% of total proteins) were identified in the *D. citri* chorion—nucleic-acid-binding, odorant-binding, and chitin-binding proteins. The chitin binding proteins included protein obstructor-E-like (27 kDa) and an uncharacterized protein (51 kDa). Protein obstructor-E-like has been reported to be critical in *Drosophila* larval development, and mutants lacking this gene failed to metamorphose correctly [57]. Protein obstructor-E directs cuticle formation during the pupation of holometabolous insects, shifting the body shape from a longitudinal (larval) arrangement to a more lateral (adult) orientation [57]. Whether this gene applies to insects without a larval stage remains to be demonstrated but offers an interesting challenge.

A ubiquitous nucleic acid-binding protein identified as zinc finger homeobox protein 3-like (85 kDa) has been found in the chorion of *D. citri*, as was odorant-binding protein-1 (16 kDa), which is common to many insects. The identification of odorant-binding proteins (OBP) in our sample, which is considered a chemoattractant for sperm in *Anopheles gambiae* and *Aedes aegypti* [51,54], suggests that the chorion of *D. citri* is important for fertilization and, therefore, is an excellent silencing target to prevent gamete fertilization.

### 3.3. Structural and Protection Proteins

Two proteins, vegetative cell wall protein gp1-like (52 kDa) and a 39 kDa uncharacterized protein, were assigned as structural proteins (8% of total proteins). The gp1-like cell wall protein is the product of a gene primarily found in plants, but it was also identified in five hemipterans. Cuticle protein 7-like (protection function) was identified in the chorion, and interestingly, more than 800 cuticle proteins have been identified in insects as a major component of their main body covering [58]. Although we found no literature existing specifically about cuticle protein 7-like, it and the gp1-like protein present opportunities for further study.

### 3.4. Vitellogenins

Several proteins identified as homeostasis-related proteins (8%) in the chorion could be possible targets to control *D. citri* by RNAi. We identified two vitellogenins that functioned as a nutritional source of eggs (170 kDa), as well as for lipid transport (64 kDa), and two vitellogenins associated with the Von Willebrand factor domain (VWFD) that are required for normal homeostasis. Vitellogenin (Vg) is a precursor protein that produces the major storage protein, vitellin, in insect eggs [59]. Several Vg genes have been silenced by RNAi in *Bemisia tabaci* [60], *Rhodnius prolixus* [39], and *Nilaparvata lugens* [61], causing a significant decrease in survival rate, increased mortality, reduction in egg count, and distorted egg-laying patterns, suggesting that Vg genes are promising targets for pest control. Vg protein was also found in the body and head of white-backed planthoppers, and its gene expression was highest in mature females, suggesting an important role in reproduction [62].

### 3.5. Gene Expression Regulation Proteins

Two proteins identified as Golgin subfamily A member 4-like (180 and 192 kDA) were detected by LC-MS/MS of *D. citri* choria. Miao et al. [62] reported the presence of golgin subfamily genes in the salivary proteome of the plant hopper, *Sogatella furcifera*. Its biological function was assigned as a Golgi transporter. A third low-quality cytoskeletal protein, myosin-II-like (non-muscle) (151 kDa), was found in the sample and is thought to be active during the mitosis and invagination of eggs during *Drosophila* embryogenesis [63].

### 3.6. Immune Response

Two proteins related to immune response (6% of total proteins) were identified, including leucine-rich repeats and immunoglobulin-like domains protein 1 (LRIG1) (60 kDa) and a 10 kDa uncharacterized protein. LRIG1 is thought to interact with epidermal growth factors during oogenesis in mammals, but its study in insects is incomplete [64]. However, in mammals, its immune response relates to the wound healing of the epidermis and suppresses cell proliferation in embryonic tissues [65].

### 3.7. Other Proteins and Uncharacterized Proteins

Two cellular process proteins (16% of total proteins), ubiquilin-1 (39 kDa) and the RNA polymerase-associated factor (Paf 1) CTR9 homolog (158 kDa), were identified and classified in the “others” category of Figure 1B. A similar ubiquilin protein was identified in *B. tabaci* fed a high phenylpropanoid/flavonoid diet. Up-regulation of this gene indicated an adaptation in *B. tabaci* for the detoxification of plant phenolics [66]. The RNA polymerase II-CTR9 homolog is present ubiquitously in all organisms and regulates DNA transcription to mRNA at the SH2 domain. It is essential for embryonic development, particularly the histone H3 trimethylation of lysine [67]. In *Drosophila*, the Paf1 homolog *CTR9* is localized to the germ cells and maturing eggs, and knock-out results in aberrant polyploid morphology of the nuclei [67]. These defects eventually became lethal to embryos and early-stage larvae.

Interestingly, 21% of identified proteins were uncharacterized but were assigned a predicted function. Two of the predicted functions included environmental challenges and the calcium-dependent carbohydrate recognition domain (CRD) (Table 1). The uncharacterized genes with CRD-predicted function could be involved in plant lectin recognition as a part of the innate immune system of *D. citri* [68]. Although there were a large number of hypothetical proteins, the genes encoding these proteins could be subjected to silencing using RNAi in order to identify their biological function in *D. citri*.

## 4. Conclusions

Eggs are highly vulnerable when they are deposited on leaves because they are exposed to environmental conditions, predators, and infections [69]. In addition, Ammar et al. [70] showed that nymphs of *D. citri* acquire the bacterial pathogen of HLB at a higher rate than adult *D. citri*. Therefore, interrupting the lifecycle at the earliest stages should have the greatest impact on the transmission of the HLB pathogen. Silencing these chorion-related genes in the early stages of choriogenesis would be advantageous to reduce the viability of the eggs after oviposition, likely reducing the hatching rate. The proteomic analysis of recovered *D. citri* choria allowed us to identify its constituent proteins and provide promising new targets for further investigations of *D. citri* control through RNAi.

## Figures and Tables

**Table 1:** Proteins identified from *Diaphorina citri* chorion.

Identified Protein	Accession Number	M.W. (kDa)	Peptides	Coverage (%)	Biological Activity
Vitellogenin-1-like VWFD domain	A0A1S3DT76_DIACI	63	(R)SEKEDPAMQIAAEAMWGENAQSGAK(I) (R)KQYIANHPQAEQCR(K) (R)LMVVEQGNQLCFSTK(A)	11.10	Homeostasis
Vitellogenin-2-like VWFD domain	A0A3Q0J6A6_DIACI	52	(R)ASSAIPSTPSK(N) (R)SEKEDPAMQIAAEAMWGENAQSGAK(I) (R)KQYIANHPQAEQCR(K)	10.80	Homeostasis
Vitellogenin-A1-like VTG domain	A0A3Q0JK51_DIACI	170	(R)TLAALHDVADQYTGTIIK(A) (R)NQDSVLAWVTNAR(H) (K)NLQVSQNLPTWELNIIK(S) (K)GSNGDLIDIIK(T) (K)FTILAGVIR(T)	4.44	Source of nutrients
Vitellogenin-likeLOC103523837	A0A1S4ERJ7_DIACI	64	(K)IAQMLNIK(R) (K)YFQVNIPIK(A)	2.94	Lipid transport
LOW QUALITY PROTEIN: probable chitinase 10 GH18 family	A0A3Q0JIK7_DIACI	306	(K)KESCAPGLHWNK(V) (R)SFLICSHGNLLK(Q) (K)QSCGPSLLWNAK(K) (R)IGPYAYSDNQWVGYDDVDMIR(T) (K)SMNLGGGMIWALDLDDFK(N) (R)AAFNPHDLLLSAAVSPSK(A) (R)GFLAYYEICDK(I)	3.84	Glycosyl hydrolases
Histone acetyltransferase p300	A0A1S4EH08_DIACI	30	(K)TNGPHIYSAQATQYSGPQQTWADLQSSPVVILVK(N) (K)LLLQNNDK(V) (K)VGILYPTNAFPVNSFPDFPVGNLILPQDMSLSK(I)	27.60	Transferase activity
Cluster of golgin subfamily A member 4-like LOC103519497	A0A3Q0JE54_DIACI	180	(R)LEAETNLEKSLEQKNR(E) (R)SNNEIDLDMELVIDKFKALQTQMK(T)	2.59	Gene expression regulation
Golgin subfamily A member 4-like LOC103510159	A0A3Q0IWE5_DIACI	192	(K)VGILYPTNAFPVNSFPDFPVGNLILPQDMSLSK(I) (R)SNNEIDLDMELVIDKFKALQTQMK(T)	0.96	Gene expression regulation
Endochitinase LOC103505867 GH18 family	A0A3Q0IKI3_DIACI	68	(R)GNWAGFADVHSPLYK(R) (R)SFTLSSGNNNYNIGTYINK(E) (K)MSVCDWPEKVDVTR(C)	8.00	Glycosyl hydrolases
LOW QUALITY PROTEIN myosin-II-like LOC103524911	A0A3Q0IIF7_DIACI	151	(R)SDSLTNVLNTTSSLNSSSLSNRSLNSSGLSNR(S) (R)ITRVTNDAPIMEVNSNSLGR(R)	3.91	Gene expression regulation
Epoxide hydrolase 4-like LOC103509850	A0A1S3D296_DIACI	46	(K)VKDERENAPTCLVDNSFGQHLYVK(L) (K)QVNQQTGLVGKMYGAVSNTVK(Y) (K)QVNQQTGLVGKMYGAVSNTVKYGNHYK(M)	12.80	Hydrolase activity
Dynein heavy chain 2, axonemal-like LOC103509704	A0A3Q0IU47_DIACI	538	(K)SETVKDLGKCMAYLVVITNCSDSLDYK(S) (K)DLGKCMAYLVVITNCSDSLDYKSLAR(M) (K)VIVEQMKVEMEENQVLVENYKK(E)	1.14	Microtubule-based movement
Odorant binding protein 1	A0A2Z2GYV0_DIACI	16	(K)CKTELNSPPEAVALLGAK(S) (K)TELNSPPEAVALLGAK(S)	12.20	Odorant binding
Voltage-dependent calcium channel VWA domain LOC103507125	A0A1S4E906_DIACI	88	(K)EGKLMVSVSTPVFDK(R) (K)LERHSHFCDKSLVQSLVFDAMVTEGLEHPSAAYLK(G)	6.54	Homeostasis
Cuticle protein 7-like LOC103507855	A0A1S3CYX2_DIACI	15	(K)YDFAYDVADSYTGDIK(S) (R)DGDYVSGYYSLVEADGSK(R) (R)IVEYTADGYNGFNAVVK(K)	36.70	Protection
Carboxypeptidase D-likeLOC103513394	A0A3Q0J1P3_DIACI	145	(K)FLVAAAQQNPSK(V) (R)DLWALQISR(N) (R)SVMKVDSIVK(G)	2.38	Carboxypeptidaseactivity
Vegetative cell wall protein gp1-like LOC113466980	A0A3Q0IQY0_DIACI	52	(R)IDNDALQSMAAANINAEEVKATGVELLNK(G) (R)QRKPRSAPSNSPGGSNVR(G)	9.61	Structure
Protein obstructor-E-like LOC103517319	A0A1S3DGK4_DIACI	27	(K)NSYYPDSIQCDLYYHCSDGQLVEEK(L) (R)CDTNVNVECGER(T)	15.00	Chitin-binding
Wiskott-Aldrich syndrome protein family member 2 LOC103507255	A0A3Q0INT8_DIACI	22	(K)CITTAEYNPQCGTDGQDYSNPGR(V) (K)VVEIEFPGVCK(S)	15.70	Protease inhibitor
Alpha-mannosidase LOC103513787	A0A3Q0J7L7_DIACI	120	(R)EQASLFSQMGYDGFMFGR(Q) (R)SSGAYIFR(N)	2.46	Glycosidase activity
Glutamic acid-rich protein LOC103510883	A0A1S4EDL9_DIACI	101	(K)EKGNDSQSSLTRK(G) (K)SQNKRESAEILEK(E)	2.88	Other
Aminopeptidase LOC103519822	A0A3Q0JIL4_DIACI	92	(K)EIMDSWTLQTGYPIVDVTR(E) (K)KENGEIELNSRDEKPIVSGGGGSGNTR(N)	5.75	Aminopeptidase activity
Leucine-rich repeats and immunoglobulin-like domains protein 1 LOC103522720	A0A1S3DQ23_DIACI	60	(K)TQCQIFGLNSTLRIYLEGNPVLCDDSMR(A) (R)IYLEGNPVLCDDSMRAVIDAMETINNNTK(I)	7.89	Immune response
Apoptosis-inducing factor 1, mitochondrial LOC103519937	A0A1S4ENS9_DIACI	81	(R)SSAAQEESTKTTAVTK(V) (R)RVEHHDHAVVSGRLAGENMTGAHK(H)	5.45	Oxyreductase activity
Ecotropic viral integration site 5 ortholog-like LOC103504936	A0A3Q0IJ13_DIACI	81	(R)HLRSLVEANHNSCQSSMDEASLSK(E) (R)LKVIEVETRADDVNPDK(K)	5.86	GTPase-activity
Ubiquilin-1 LOC103506324	A0A3Q0ILW3_DIACI	39	(R)ALSNLESIPGGYSALQR(M) (R)DIQEPMLNAATQQFSR(N)	9.35	Cellular processes
RNA polymerase-associated protein CTR9 homolog LOC103509140	A0A3Q0ISU5_DIACI	158	(K)EEYFLKANTLYTTGDK(I) (K)AQPGNYETMKILGSLYANSSSQSKR(D)	2.95	Cellular processes
Chitooligosaccharidolytic beta-N- acetylglucosaminidase-like LOC103512791	A0A3Q0J0G8_DIACI	38	(K)LLDQTSLNISNNPELK(S) (K)SLIMGQEAALWSEQADAATLDGR(L)	11.70	Hydrolase activity
Protein BTG2-like LOC103513815	A0A3Q0J2K8_DIACI	24	(-)MQDQISAAVLFLAK(L) (K)KDTILENAAKAVGMSYEDMR(L)	16.30	Other
Zinc finger homeobox protein 3-like LOC113472870	A0A3Q0JJ91_DIACI	85	(R)LLMLVDQSHWLNTVSRPSNANQQSSSESSSASKHEK(S) (K)KMIQDILTGSSAVQK(S)	6.86	Nucleic acid-binding protein
Alpha-galactosidase LOC103508314	A0A1S4EAR0_DIACI	17	(R)CNTDCDNYPDECISENLFK(R) (R)CNTDCDNYPDECISENLFKR(M)	13.70	Hydrolase activity
Uncharacterized protein LOC103507240	A0A1S3CXQ3_DIACI	16	(R)DFPGMCFASTK(C) (R)LLELVEDCGPLPK(A) (K)TAPFPDCCPTFECEPGVK(L)	43.40	Environmental challenges
Uncharacterized protein LOC103519250	A0A1S3DJY7_DIACI	44	(K)NESSNGSTVNSPVAQAKPK(G) (K)GLAVAGEGGVADSSPSGTALVGK(K)	10.30	Uncharacterized
Uncharacterized protein LOC103508449	A0A1S3CZS7_DIACI	15	(R)RNLPNAYNLCEPETQVCTR(Y) (R)NLPNAYNLCEPETQVCTR(Y) (K)QATAICGPSYVDKLVDFK(T)	27.60	Uncharacterized
Uncharacterized protein LOC103512175	A0A1S4EEU2_DIACI	38	(R)GSYSYVAPDGTPIK(V) (R)GSYSYVAPDGTPIKVVYTSGR(E)	5.80	Structure
Uncharacterized protein LOC113467831	A0A3Q0IZD8_DIACI	10	(K)FAQDCDADGQIDCR(D) (R)LGGYGCNAPLDATYLAR(F)	33.00	Immune response
Uncharacterized protein LOC103507653	A0A1S3CZP5_DIACI	56	(K)GNQVNGVRENEDLIDVTSNHVNK(L) (R)AQMIYLQTELASAQNSGILPFDDSAK(N)	9.92	Uncharacterized
Uncharacterized protein LOC103513825	A0A1S3D966_DIACI	11	(R)QAQNTQLIPQGAEIQKVQEGIELAAFESIPGNQR(I) (K)VQEGIELAAFESIPGNQR(I)	34.00	Uncharacterized
Uncharacterized protein LOC103518044	A0A3Q0JFF5_DIACI	428	(R)AIGQDCTVTK(S) (R)QESIQGFCCPR(Y) (K)QLGCLVEGQTYMVGEK(V)	0.91	Homeostasis VWFC domain
Uncharacterized protein LOC103505974	A0A3Q0IQQ7_DIACI	91	(K)DTEVDRVQK(S) (K)QLDQAAKEIEELKSENQIMK(R)	3.64	Peptidase activity
Uncharacterized protein LOC103513107	A0A1S3D7M4_DIACI	136	(K)VDNSPNRTQYGELLK(T) (R)QNTINIAKLGGIESPEKDELK(N)	3.00	Uncharacterized
Uncharacterized protein LOC113468219	A0A3Q0IX19_DIACI)	113	(K)CVKTTKMLPGGQK(K) (K)FKSRGLLGDNGFQSGGLLDDK(D)	3.43	Uncharacterized
Uncharacterized protein LOC103506631	A0A3Q0ISV1_DIACI	30	(K)IVTSTGGLPPQFVNPK(S) (R)SQVCIAHGPYAAIPGINEWCETNCLR(F)	15.60	Uncharacterized
Uncharacterized protein LOC103512422	A0A1S3D6F7_DIACI	22	(R)SLEVDWLDAR(N) (R)NTGDWSATGGFGQAQPDNR(E)	14.70	Calcium-dependent carbohydrate-recognition domain
Uncharacterized protein LOC103516159	A0A1S3DEF2_DIACI	12	(K)EVTQLASSIAPFSDPTICDDPFKCDEDTIIPDTICGK(L) (K)LYTTAGNGLR(I)	43.90	Uncharacterized
Uncharacterized protein LOC103516900	A0A1S3DFS2_DIACI	51	(K)DVEFVCPDEGGNGNYADPSTCR(R) (K)SETAPLEACNLPNK(C)	7.95	Chitin binding
Uncharacterized protein LOC103519755	A0A1S3DJA9_DIACI	36	(K)VAKPSYVVQEQVIPVQKPAYVIEEAVEK(V) (K)VTKPAFVVETIVETVPVPEFK(L)	14.90	Uncharacterized
Uncharacterized protein LOC103506731	A0A3Q0ISB2_DIACI	21	(R)SIGLQLTSFESR(E) (K)SDSITQYLTNAGYNK(Y)	14.60	Calcium-dependent carbohydrate-recognition domain
Uncharacterized protein LOC103509128	A0A3Q0IST9_DIACI	233	(R)STDGKNQRPSALPSAMAPNKLDSDR(R) (R)QIHVMLHAFIREGGAVLQMQQESR(L)	2.34	Actin binding
Uncharacterized protein LOC103509950	A0A3Q0IUI2_DIACI	86	(K)SDFVQNDGSAGKFGAGDSRSK(D) (R)TPLERTSASGASQSPMSINKATNINR(M)	5.84	Uncharacterized
Uncharacterized protein LOC103512184	A0A3Q0J4A1_DIACI	149	(K)NQVMYKPDIPSGGSLSSLPIYAEVNK(K) (K)VKDIDQLSTTSNNSNINVFIVNKR(S)	3.72	Uncharacterized

## Data Availability

Data will be shared upon request to the corresponding authors.
